# Pharmacological applications of natural peptide libraries

**DOI:** 10.1186/2050-6511-13-S1-A31

**Published:** 2012-09-17

**Authors:** Johannes Koehbach, Markus Muttenthaler, Carsten Gründemann, Christian W Gruber

**Affiliations:** 1 Center for Physiology and Pharmacology, Medical University of Vienna, 1090 Vienna, Austria; 2 Departments of Chemistry and Cell Biology, The Scripps Research Institute, La Jolla, CA 92037, USA; 3 Complementary Medicine, Department of Environmental Health Sciences, University Medical Center Freiburg, 79111 Freiburg, Germany

## Background

The diversity in nature has long been and still is one of the biggest resources of pharmaceutical lead compounds and many natural products often exhibit biological activity against unrelated biological targets, thus providing us with starting points for drug development. Natural peptides of great number and diversity occur in all organisms from plants to microbes to man. Examples for such rich and yet largely untapped libraries of bioactive compounds are animal venom peptides, insect peptide hormones or plant defense peptides [1]. Our goals are (i) to discover and characterize novel bioactive peptides, (ii) to screen their pharmacological activity *in vitro*, (iii) to synthesize optimized peptide compounds and (iv) to determine their potential as pre-clinical drug candidates.

## Methods

As proof-of-concept we have used a genome-mining approach or mass spectrometry and peptidomics to determine the occurrence and molecular structure of naturally-occurring peptides and have investigated their pharmacological profile on human oxytocin and vasopressin receptors, representative members of the GPCR family, as well as their anti-proliferative activity on cells of the human immune system. Circular plant peptides have been identified as potent immunosuppressive agents and promise great potential as templates for pharmaceutical applications due to their enormous stability and sequence diversity [2]. On the other hand we are exploring the pharmacological potential of endogenous insect vasotocin-like peptide hormones and marine cone-snail venom peptides as receptor-subtype selective ligands for the treatment of a wide range of challenging, but yet untreatable diseases.

## Discussion

Unlike small molecules, peptides are just at the beginning as potential drug sources and still face a range of significant drug development challenges including efficient drug delivery, oral bioavailability and central penetration. Nevertheless, the ease of synthesis, the vast natural abundance of bioactive peptides and their immense pharmacological potential are so convincing that it is just a question of time until peptides can be utilized as orall bioavailable or even CNS-penetrating drugs together with an efficient delivery platform preserving their selectivity and interaction with extracellular targets, yet simultaneously retaining stability to enzymatic degradation.
